# Analysis of biomechanical load cases for the investigation of pedicle screws ASTM F1717 vs. Toggling

**DOI:** 10.3389/fbioe.2026.1901026

**Published:** 2026-08-28

**Authors:** Stefan Schleifenbaum, Lisa Marie Tiesler, Florian Metzner, Philipp Pieroh, Christoph-Eckhard Heyde

**Affiliations:** 1 ZESBO - Center for Research on Musculoskeletal Systems, Faculty of Medicine, Leipzig University, Leipzig, Germany; 2 Department of Orthopaedic Surgery, Traumatology and Plastic Surgery, University of Leipzig Medical Center, Leipzig, Germany

**Keywords:** ASTM F1717, biomechanic spine, comparative study, screw testing, toggling

## Abstract

**Background:**

The anchorage of pedicle screws is essential for spinal stability during surgical procedures. Static pullout tests differ substantially from the dynamic loading conditions that occur *in vivo*. This study aimed to compare two dynamic load cases (ASTM F1717 and Toggling) to assess the implementation of the test results to clinical failure cases as well as their implications for standardization.

**Methods:**

The two loading cases were investigated under standardized conditions using a 6DoF sensor and two types of polyurethane foams. Both test configurations were examined on an adaptable test setup that allowed the integration of the 6DoF sensor and the foam blocks (10 PCF Blocks from Sawbones of the following types: open-cell and cellular foam). Measurements were performed under compression (10–110 N) and under alternating compression and tensile load (±110 N). Cyclic loading was applied for 15 cycles when using the 6DoF sensor and for 500 cycles when the screws were anchored in the foam.

**Results:**

In the ASTM F1717 setup, the boundary conditions resulted in a significantly higher force in the z-direction and a positive moment around the test bench’s rotational axis. In the Toggling setup, an alternating moment around the rotational axis and a displacement in the z-direction were observed. Most foam (100% open-cell foam and 50% cellular foam) reached the failure criterion before completion of testing. The cellular foam withstood the ASTM F1717 loading in both directions, whereas under Toggling, it completed 40 ± 18 cycles in compression and 11 ± 5 cycles under alternating compression and tensile loading.

**Conclusion:**

The 6DoF measurements yielded consistent results, which were determined by the specific boundary conditions of the two load cases. However, the isolated load cases used in ASTM F1717 and in Toggling tests do not reflect the full spectrum of physiological loading. For the development of a universal testing method for pedicle screws in human specimens, the implementation of a multiaxial cyclic loading should be considered. In the future, universal spine simulators could be alternative to the isolated loading protocols currently in use.

## 1 Introduction

Secure pedicle screw anchorage is essential in spine surgery. In the treatment of unstable spinal segments, fractures, or fusion procedures, the stability and durability of screw fixation are key determinants of successful clinical and long-term outcomes (Schatzker et al., 1975; Bydon et al., 2015; Wu et al., 2019; Wu et al., 2025). The choice of fixation technique depends on factors such as the clinical indication, bone quality, and patient-specific anatomy, as well as the need for deformity correction or reduction. Various screw designs, anchoring techniques, and fixation strategies have been developed to optimize bone purchase and enhance construct stability (Zindrick et al., 1986; Bydon et al., 2015; Matsukawa et al., 2021). Despite these efforts, screw loosening remains a major factor contributing to revision surgery (Wu et al., 2025). The literature reports loosening rates ranging from 1% to 60% (Da et al., 2020; Marie-Hardy et al., 2020; Lai et al., 2022; Xu et al., 2022), which then lead to corresponding revision surgeries.

In general, pedicle screw anchorage is biomechanically evaluated with static pullout tests as the most widely used method, measuring the maximum axial extraction force a screw can withstand. These tests are easy to perform, well standardized, and provide quantitative data on fixation strength (Karami et al., 2015; Goda et al., 2016). But so far, those represent a static loading condition and fail to reflect the dynamic and complex environment vivo loading (Karami et al., 2015; Goda et al., 2016).

In a clinical setup, spinal implants are exposed to multidirectional, cyclic loads induced by movement, vibration, and repetitive activity (Rohlmann et al., 1997; 1999). Previous studies have shown that screws perform markedly differently under dynamic loads and static pullout conditions (Kueny et al., 2014; Murray, 2014; Schulze et al., 2020; Jarvers et al., 2021; Schleifenbaum et al., 2023). Cyclic loading leads to greater mechanical stress at the bone–screw interface and might cause fatigue, loosening, or even failure. Hence, evaluating anchorage stability under dynamic conditions is essential to better understand implant performance under physiological loads (Murray, 2014; Schleifenbaum et al., 2023; Schleifenbaum et al., 2025). For this reason, it is important to better understand the load protocols used in order to be able to compare studies.

According to Murray, conceivable load cases of pedicle screws are categorized into ASTM F1717, moment, and Toggling protocols (Murray, 2014). These protocols differ with respect to their test setups and boundary conditions during testing. In the ASTM F1717 configuration, rotation about two axes is permitted, whereas the other two load cases allow compensatory motion within the horizontal plane. The moment and the Toggling protocol differ primarily in the displacement of the axis of rotation. In the moment protocol, the axis is centered above the specimen and is transmitted to the screw via a lever arm. In the Toggling protocol, the axis of rotation is located directly at the screw head. In both cases, the load is generated by axial displacement. The terms ‘moment protocol’ and ‘Toggling protocol’ are often used interchangeably. However, the ASTM F1717 and Toggling testing are the most established test methods.

Depending on the specific test arrangement, loading is predominantly introduced either as a moment or as an axial force via the screw-rod interface. Consequently, the comparability of studies conducted using the ASTM F1717 (Murray, 2014; Jarvers et al., 2021; Schleifenbaum et al., 2025) or Toggling protocols (Murray, 2014; Bostelmann et al., 2017; Schmoelz et al., 2018; Schmoelz and Kienle, 2023) is limited, as key parameters such as load mode, loading frequency, and number of cycles vary between investigations. Furthermore, the literature indicates that there is no standardized terminology regarding the protocols used. The main challenge is developing reproducible test methods that simulate realistic physiological loading while enabling meaningful comparisons across studies (Dreischarf et al., 2016; Spicher et al., 2023).

The present study aims to investigate and compare biomechanical testing methods for pedicle screws under dynamic loading conditions. The comparison of these load cases focuses on their reproducibility and their ability to represent clinically relevant conditions. By analyzing the test outcomes, this study aims to contribute to the standardization of biomechanical testing methods for pedicle screws. Improved standardization will enhance the comparability and interpretability of results across studies and, ultimately, support the development and selection of spinal implants that provide stable, durable fixation and improved patient outcomes.

## 2 Materials and methods

### 2.1 Setup

**FIGURE 1:**
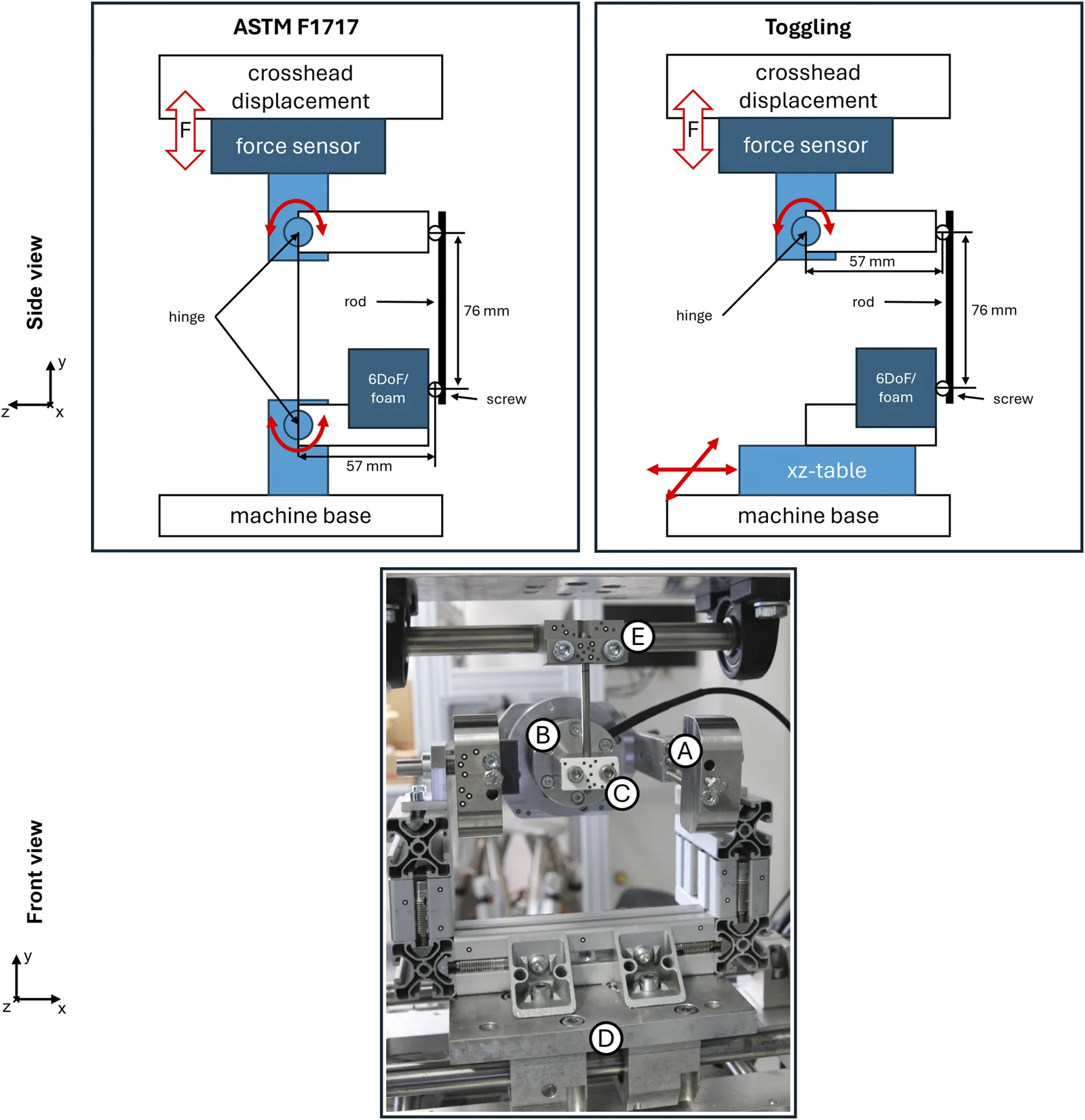
Test setup top: schematic description of the boundary conditions of ASTM (left) and Toggling (right); bottom: both load cases can be applied in a combined test setup consisting of: **(A)** rocker balanced using counterweights, **(B)** 6DoF sensor, **(C)** lever arm 57 mm and lower rod clamp, **(D)** substructure and xz-table, **(E)** upper axis of rotation with lever arm 57 mm and upper rod clamp.

The two load cases, ASTM F1717 (ASTM - American Society for Testing and Materials) and Toggling, were compared using cyclic testing. A custom test setup was designed that enabled the application of both load cases. A lockable rocker (Figure 1, bottom A) served as the mount for a six-degrees-of-freedom (6DoF) force–moment sensor (Figure 1, bottom B; K6D40 (Fmax_x_ = 500 N, Fmax_y_ = 500 N, Fmax_z_ = 2000 N, accuracy class = 0.2, sampling rate = 5 Hz) ME-Meßsysteme GmbH, Hennigsdorf, Germany). The rocker can be locked in place with a pin connecting the frame and the rocker, preventing rotation during Toggling. Depending on the load case under investigation, the boundary conditions could thus be adjusted within the same test setup. The free mounting side of the sensor was positioned and fixed at the height of the rocker’s axis of rotation using a 3D-printed device (PLA using Fused Deposition Modeling). A lever arm of 57 mm was attached to the sensor and connected to the lower rod clamp (Figure 1, bottom C). Before testing, the rocker was balanced using counterweights on both sides. The base of the test rig was mounted on an xz translation table (Figure 1, bottom D). The vertical load was applied by the crosshead displacement of the universal testing machine (AllroundLine Z10, Zwick/Roell GmbH and Co. KG, Ulm, Germany). A shaft with a lever arm of 57 mm was attached underneath the crosshead, which introduced the load into a straight rod system (M.U.S.T.; Medacta International SA, Switzerland) (Figure 1, bottom E). The rod was fixed between the centers of both clamps with a clamping length of 76 mm.

### 2.2 Testing procedure

**FIGURE 2:**
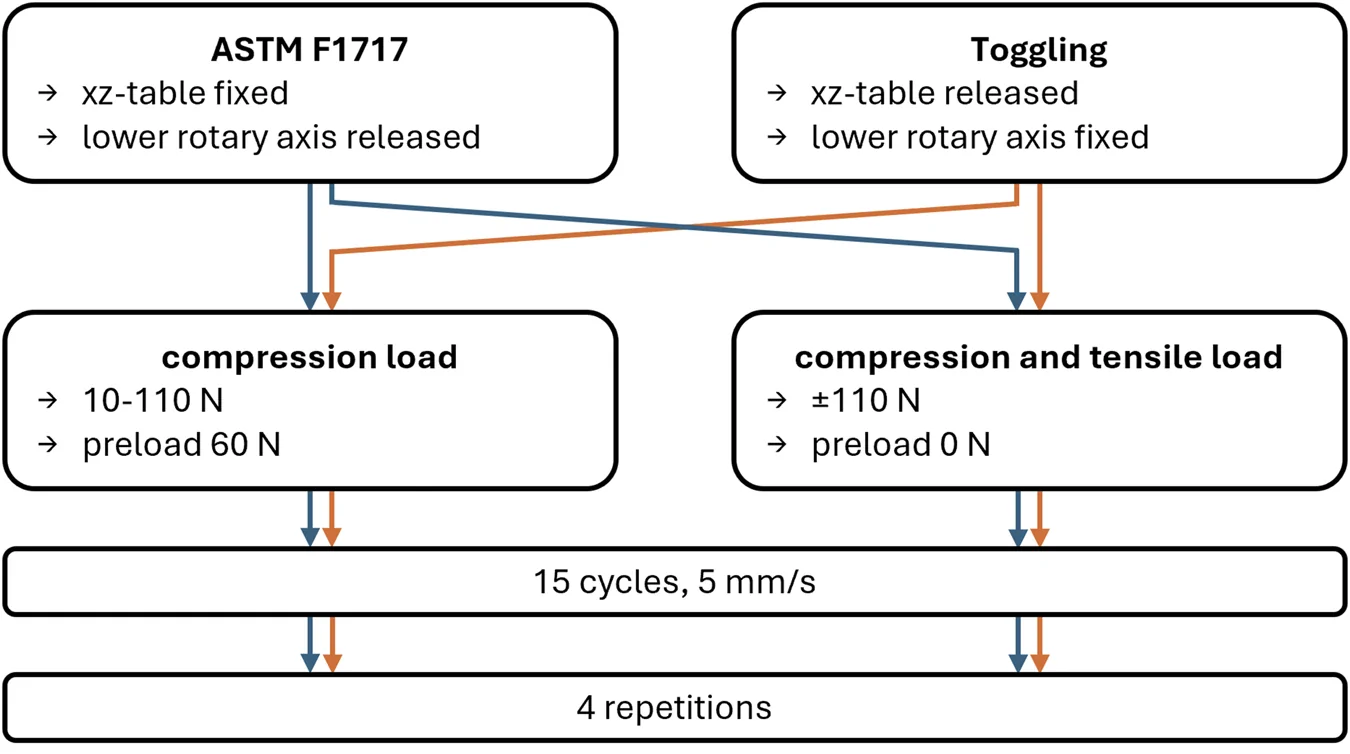
Flow diagram of the test sequence differentiated according to load cases and load directions.

Both load cases were tested in two loading directions using a universal testing machine. The specific boundary conditions for each test are shown in Figure 1 (top), Figure 2, Table 1:

- ASTM: For testing according to ASTM F1717, the xz-table was fixed while both rotational axes were released, allowing movement of the lower rig in only one degree of freedom (Figure 1, top-left). Cyclic compressive loading between 10 and 110 N was applied for 15 cycles at a crosshead speed of 5 mm/s. The crosshead speed was set for comparison purposes, in line with previous Toggling investigations (Schmoelz et al., 2018). A preload of 60 N was applied before measurement. Additionally, alternating compressive and tensile loads of ±110 N were applied for 15 cycles at 5 mm/s without preload.
- Toggling: For the Toggling tests, the xz-table was released, and the lower rotational axis was fixed, allowing movement of the lower rig in two degrees of freedom (Figure 1, top-right). Compression loading between 10 and 110 N was applied for 15 cycles at 5 mm/s with a 60 N preload. Furthermore, cyclic alternating compressive and tensile loads of ±110 N were applied for 15 cycles at 5 mm/s without preload.

**TABLE 1:** Boundary conditions of the four load cases during cyclic loading for 15 cycles at 5 mm/s crosshead speed with four repetitions using a 6DoF force-moment sensor.

Load cases	ASTM F1717	Toggling
Boundary conditions	xz-table fixed, lower rotary axis released, two DOF	xz-table released, lower rotary axis fixed, one DoF
Compression	load: 10–110 N (preload 60 N)	load: 10–110 N (preload 60 N)
Compression and tension	load: ±110 N (preload 0 N)	load: ±110 N (preload 0 N)

The force was selected based on *in vivo* data from Rohlmann et al. (1999). The force exerted by the instrumented screw-rod system was 220 N along the longitudinal axis. In our study, only one side of the screw-rod system was tested, so the force was halved to a maximum of 110 N. The resulting load range for the compression test was compared with that specified in ASTM F1717 (ASTM F04 Committee, 2015). This resulted in a load range between 10 N and 110 N. To ensure comparability within the compression range, the 110 N value was also selected for alternating compressive and tensile testing. This resulted in a load range between −110 N and 110 N.

For each of these four test sequences, four repeated trials were performed. The failure criterion for the cyclic test was defined as exceeding a maximum displacement of 20 mm relative to the starting position, at which point component collision would occur.

The motion of the lower rod was recorded using a marker-based 3D optical measurement system (ARAMIS 3D, Carl Zeiss GOM Metrology GmbH, Braunschweig, Germany) at a sampling frequency of 5 Hz and a measurement accuracy of 0.015 pixel (Schroeder et al., 2022). Comparison of the two load cases and directions was based on cycles 6–10 of each measurement, yielding a total of 20 cycles per loading condition (ASTM vs. Toggling) for statistical evaluation. Displacement and rotation of the lower rod relative to the rocker were analyzed.

### 2.3 Foam testing

Both load cases were also evaluated using synthetic bone substitutes: cellular foam (1522-10, Sawbones Inc., Vashon, WA, United States, ASTM D1621 Strength 2.3 MPa, Modulus 23 MPa, Dimension 130 × 180 × 40 mm) and open-cell foam (1522-523, Sawbones Inc., Pacific Research Laboratories, Vashon, WA, United States, ASTM D1621 Strength 0.53 MPa, Modulus 35 MPa, Dimension 130 × 180 × 40 mm), each with a density of 10 PCF (Pounds per Cubic Foot). The foams were selected based on a comparison of their material properties with those of human vertebral trabecular bone (Kopperdahl et al., 2002; Gehweiler et al., 2021; Metzner et al., 2026). In addition, two different types of foam should be selected. Each foam block was cut into 24 rectangular blocks (40 × 32.5 × 30 mm) using a bandsaw. A central pilot hole (2.5 mm) was orthogonal to the 32.5 × 30 mm surface drilled using a bench drill. The threads were then cut manually under guided threading, and the cannulated polyaxial pedicle screws (6 × 40 mm, M.U.S.T.; Medacta International SA, Castel San Pietro, Switzerland) were inserted 35 mm deep using the appropriate tools.

Five specimens per foam type were tested under each of the four previously described sequences (Table 1) over 500 cycles:

- ASTM under compression (n = 5) and alternating compression and tensile load (n = 5)
- Toggling under compression (n = 5) and alternating compression and tensile load (n = 5)

Testing was terminated when displacement from the universal testing machine exceeded 20 mm. Optical measurements were recorded at cycles 1–10, 25, 50, 100, 200, and 495.

### 2.4 Statistical analysis

Data from the 6DoF sensor were processed using MATLAB R2025a (The MathWorks Inc., Natick, MA, United States). Force and moment data were transformed, and minimum and maximum values were extracted. Data sets were merged, visualized, and the range of extreme values was calculated using spreadsheet software (Microsoft Excel v2506, Redmond, WA, United States). Graphical representations were based on mean values from repeated measurements.

Statistical comparisons between the two load cases (ASTM vs. Toggling) under compression and alternating compression and tensile loading were performed using nonparametric Mann-Whitney U tests in SPSS v29.0 (IBM Corp., Armonk, NY, United States). Statistical significance was defined as p < 0.05, and 95% confidence intervals were calculated for all mean differences. Each parameter was tested for Gaussian distribution. For three parameters—maximum Mx during Toggling under compression, minimum Fy for ASTM under compression, and the range of Fy for ASTM while alternating compression and tensile load—the Gaussian distribution was rejected, and comparisons were therefore reported descriptively.

## 3 Results

The two load cases were compared based on the forces and moments recorded by the 6DoF sensor, as well as the three-dimensional displacement of the screw head. The horizontal force along the test stand’s rotation axis was defined as Fx (along the transverse axis), the vertical force as Fy (along the sagittal axis), and the horizontal force perpendicular to the rotation axis as Fz (along the longitudinal axis). Optical motion tracking revealed negligible displacements along the x-axis due to the application of a perpendicular force in both load cases. In contrast, clear differences were observed in the main displacement direction of the screw head: during the ASTM tests, the predominant motion occurred along the y-axis, whereas in the Toggling tests, displacement along the z-axis was dominant. To ensure a consistent comparison of the diagrams of the respective data from the 6DoF force-moment sensor and the optical system, the crosshead displacement of the testing machine was chosen as a uniform basis.

### 3.1 Compression

**FIGURE 3:**
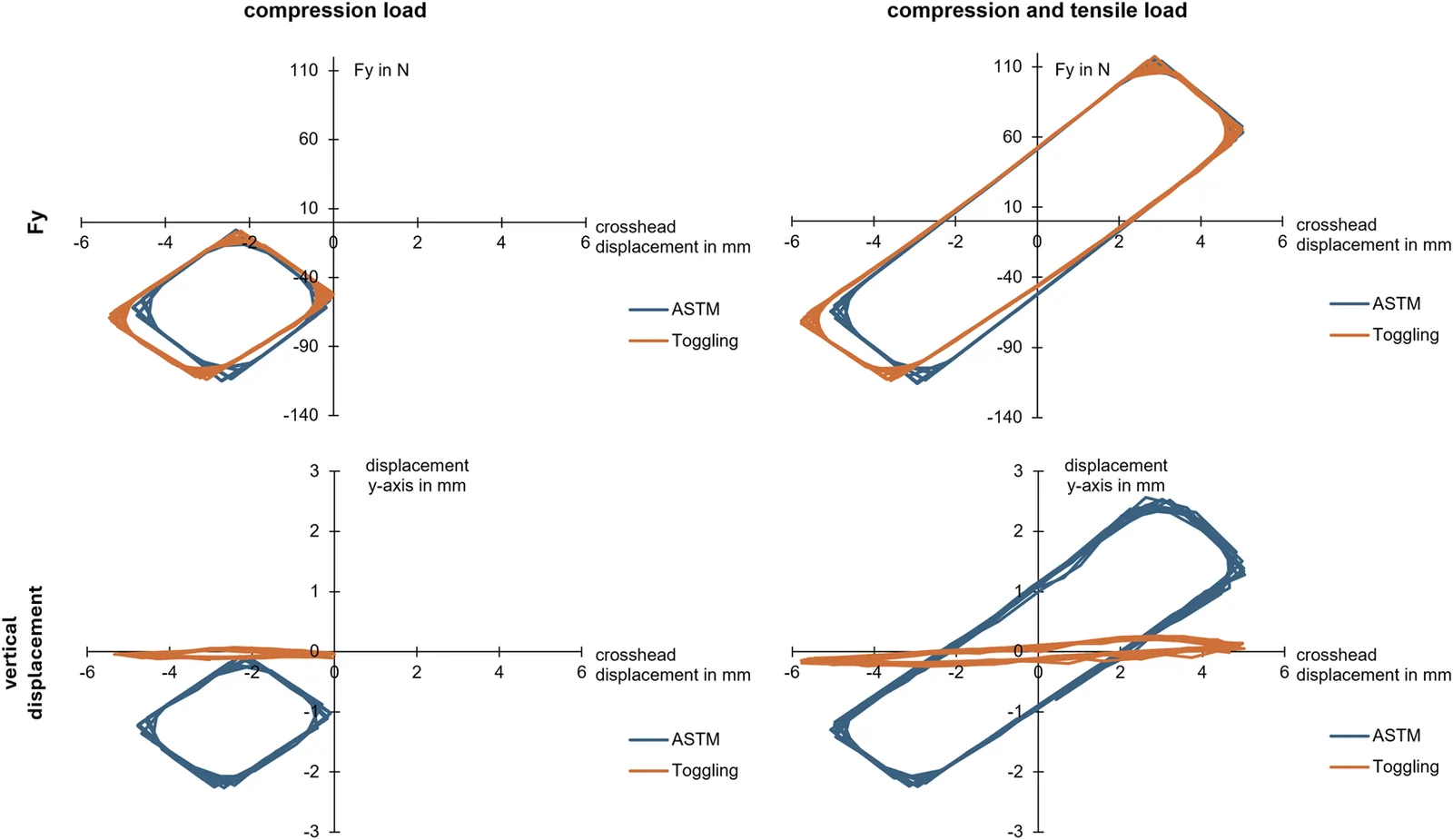
Fy (top) and vertical displacement in y-direction (bottom) during both load cases under compression (left) and alternating compression and tensile load (right) of 10 cycles.

**FIGURE 4:**
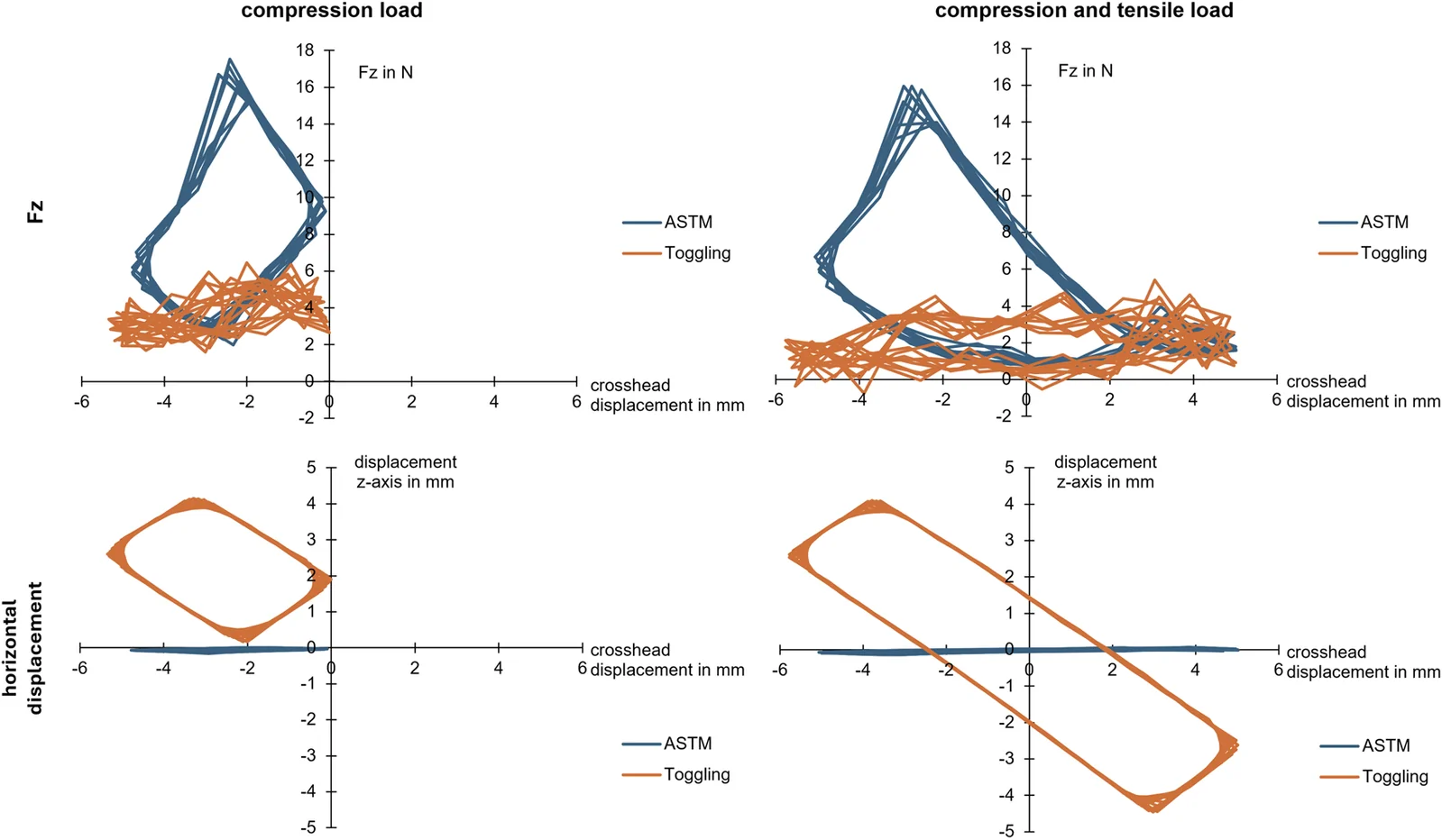
Fz (top) and horizontal displacement in z-direction (bottom) during both load cases under compression (left) and alternating compression and tensile load (right) of 10 cycles.

Under compressive loading, the Fx curves of both load cases showed similar shapes but differed in their force ranges. ASTM produced forces ranging from −7.89 N to −5.02 N, whereas Toggling generated smaller forces ranging from −1.72 N to 0.85 N, resulting in a significant difference between the extreme values (p < 0.001, Table 2). The largest portion of the applied force was detected in the Fy direction (along the longitudinal axis in the cranio-caudal direction), corresponding to the externally applied compressive force of 10–110 N (Figure 3). No significant differences in Fy were found between the two load cases. For Fz, the load cases exhibited distinct curve characteristics (Figure 4, bottom-left). Toggling generated small horizontal forces of 6.27 N, while ASTM reached 16.32 N, resulting in significant differences between the maxima (p < 0.001), minima (p < 0.001), and range (p < 0.001).

**TABLE 2:** Statistical evaluation ASTM vs. Toggling under compression.

Load cases		Minima				Maxima			
		Mean ± SD	Median	IQR	p-value	Mean ± SD	Median	IQR	p-value
ASTM	Fx (N)	−7.89 ± 0.31	−7.84	0.08	<,001	−5.02 ± 0.23	−5.03	0.12	<,001
Toggling		−1.72 ± 0.23	−1.67	0.19		0.85 ± 0.23	0.87	0.25	
ASTM	Fy (N)	−110.00 ± 3.45*	−109.91*	0.18*	0.965	−10.50 ± 3.18	−10.48	0.38	0.996
Toggling		−109.96 ± 3.44	−110.59	1.58		−10.49 ± 3.58	−11.51	2.13	
ASTM	Fz (N)	2.88 ± 0.55	2.86	0.24	<,001	16.32 ± 0.81	16.26	0.21	<,001
Toggling		1.21 ± 0.76	1.25	0.92		6.27 ± 0.67	6.24	0.40	
ASTM	Mx (Nm)	0.19 ± 0.02	0.18	0.01	<,001	0.75 ± 0.03	0.76	0.01	<,001
Toggling		−0.44 ± 0.10	−0.45	0.03		0.33 ± 0.10*	0.34*	0.02*	
ASTM	My (Nm)	−0.26 ± 0.02	−0.26	0.00	<,001	−0.18 ± 0.01	−0.18	0.01	<,001
Toggling		0.00 ± 0.01	0.00	0.00		0.07 ± 0.01	0.07	0.01	
ASTM	Mz (Nm)	0.04 ± 0.01	0.04	0.00	<,001	0.09 ± 0.01	0.09	0.01	<,001
Toggling		−0.20 ± 0.01	−0.20	0.00		−0.16 ± 0.01	−0.16	0.01	

* no normal distribution.

**FIGURE 5:**
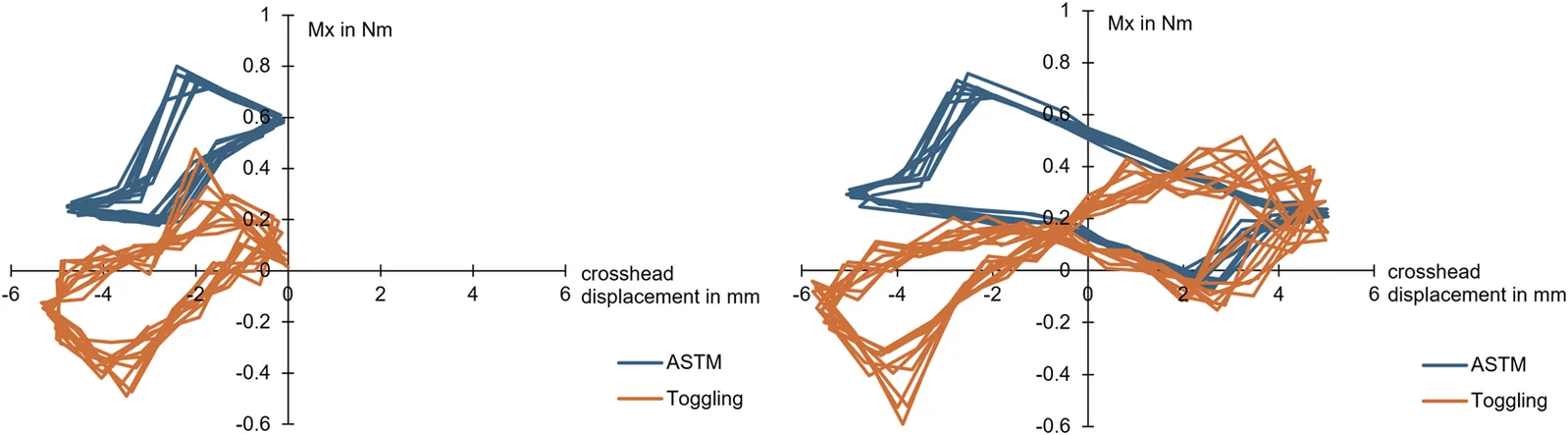
Mx during both load cases under compression (left) and alternating compression and tensile load (right) of 10 cycles.

The moment around the test stand’s rotation axis (Mx) was the key parameter distinguishing the two load cases. ASTM showed an exclusively positive moment range of 0.19–0.75 Nm, whereas Toggling induced a reversal of moment direction from −0.44 to 0.33 Nm (Figure 5, left). Consequently, significant differences were observed for the maxima, minima, and range of Mx (p < 0.001 for all). The additional moments My and Mz were comparatively small and had minimal influence on the load response.

### 3.2 Alternating compression and tensile load

When comparing pure compression and alternating compression and tensile loading, the load-displacement curves followed similar trajectories within the compressive regime. Adding tensile load resulted in a symmetric extension of the curve in the graphical representation. Fx followed comparable patterns for ASTM and Toggling; however, both load cases differed significantly in force ranges (p < 0.001, Table 3). ASTM produced purely negative forces (−7.65 to −1.05 N), while Toggling yielded bidirectional forces (−1.45–3.66 N). The alternating compressive and tensile loading of ±110 N applied by the testing machine was confirmed by both graphical and statistical analysis (Figure 3, top-right). For Fy, a significant difference between the minima of the two load cases was detected (p = 0.028). In the z-direction, ASTM again exhibited exclusively positive forces (0.47–15.02 N), whereas Toggling generated alternating bidirectional forces (−0.71–4.70 N, Figure 4 top-right), resulting in significant differences in maxima, minima, and range of Fz (p < 0.001 each).

**TABLE 3:** Statistical evaluation ASTM vs. Toggling under compression and tensile load.

Load cases		Minima				Maxima			
		Mean ± SD	Median	IQR	p-value	Mean ± SD	Median	IQR	p-value
ASTM	Fx (N)	−7.65 ± 0.21	−7.61	0.10	<,001	−1.05 ± 0.26	−1.06	0.18	<,001
Toggling		−1.45 ± 0.18	−1.42	0.07		3.66 ± 0.26	3.60	0.11	
ASTM	Fy (N)	−110.83 ± 3.69	−110.77	0.47	0.028	111.81 ± 3.50	111.84	0.64	0.355
Toggling		−108.43 ± 2.90	−108.68	2.52		110.71 ± 3.97	111.04	3.75	
ASTM	Fz (N)	0.47 ± 0.36	0.47	0.18	<,001	15.02 ± 0.76	14.99	0.31	<,001
Toggling		−0.71 ± 0.49	−0.69	0.28		4.70 ± 0.65	4.76	0.41	
ASTM	Mx (Nm)	−0.05 ± 0.06	−0.05	0.00	<,001	0.70 ± 0.02	0.71	0.00	<,001
Toggling		−0.34 ± 0.08	−0.35	0.03		0.45 ± 0.05	0.46	0.01	
ASTM	My (Nm)	−0.27 ± 0.01	−0.27	0.01	<,001	−0.08 ± 0.01	−0.08	0.01	<,001
Toggling		0.00 ± 0.01	0.00	0.00		0.14 ± 0.01	0.13	0.01	
ASTM	Mz (Nm)	−0.02 ± 0.01	−0.01	0.00	<,001	0.08 ± 0.01	0.08	0.00	<,001
Toggling		−0.18 ± 0.01	−0.18	0.00		−0.12 ± 0.01	−0.12	0.00	

Despite the alternating nature of the applied load, ASTM predominantly exhibited a positive moment (−0.05 to 0.70 Nm), while Toggling induced a sliding-like reversal of Mx. In contrast to ASTM, the maximum moment of 0.45 Nm occurred during tensile loading, with negative moments up to −0.34 Nm under compression (Figure 5, right). Significant differences between extreme points were again observed for both loading cases (p < 0.001), whereas the overall range of Mx showed no significant difference.

### 3.3 Foam for screw mounting

The cellular foam completed all cycles under ASTM loading but failed early under Toggling. Specifically, it withstood 40 ± 18 cycles under compression and 11 ± 5 cycles under alternating compression and tensile loading before reaching the failure criterion (Table 4). The open-cell foam failed prematurely in all tests: during ASTM and Toggling under compression, failure occurred already during the preload phase. Under alternating compression and tensile loading, all specimens failed within the first cycle, meeting the defined failure threshold immediately.

**TABLE 4:** Overview of which foams passed or failed 500 cycles test protocol under ASTM and Toggling.

Load cases		Celluar foam	Time of failure	Open-cell foam	Time of failure
ASTM	Compression	5/5	​	0/5	preload
	Compression and tension	5/5	​	0/5	first cycle
Toggling	Compression	0/5	≤55 cycles	0/5	preload
	Compression and tension	0/5	≤22 cycles	0/5	first cycle

## 4 Discussion

The two load cases, ASTM and Toggling, and the occurring loosening mechanisms, were analyzed by measuring the transmitted forces and moments using a 6DoF sensor and by conducting foam tests. The rapid or immediate failure of the foams indicates that these materials are poorly suited to the two investigated load cases. In particular, the open-cell foam was difficult to fix within the test setup, as it fractured already during clamping. The literature indicates that an initial load of 50 N or more is used for cyclic testing of pedicle screws (Kueny et al., 2014; Bostelmann et al., 2017; Schmoelz et al., 2018; Schulze et al., 2020). To ensure comparability between test methods and to investigate a minimum level of primary stability, the test parameters should remain unchanged.

However, it should be noted that these foams are intended as substitutes for osteoporotic bone. By using these foams, we hoped to gain insights into the loosening mechanisms at the screw-foam interface under the two load cases and directions. The narrow pedicle canal with its cylindrical cortical layer is crucial for a reliable screw–vertebrae interface. A study by Hirano et al. demonstrated that the pedicle accounts for approximately 80% of the cranio-caudal stiffness of the entire vertebral body (Hirano et al., 1997).

Nevertheless, the cellular foam exhibited differences between the two load cases. While the ASTM tests could be completed without failure, this foam failed during the first few cycles under Toggling. These findings suggest distinct loading and failure modes between the two test protocols. The failure of the foam therefore, appears to have been caused not by the alternating load direction, but by the loads acting during Toggling. The results suggest that the alternating moment Mx has a greater influence on the loosening of the foam than the loads in the Fz direction and the positive moment Mx induced by the ASTM load case. In addition to the characteristics of the porous foam structure, the Toggling load case also appears to contribute to the failure of the foam specimens.

During the measurements with the 6DoF sensor, the influence of individual specimens or foam variations was minimized by introducing the load into the sensor via a lever. Due to the released degrees of freedom, motion, as well as the corresponding reaction forces and moments, were able to develop freely. These showed plausible relationships, such as an increase in the Fz force under ASTM, since the boundary conditions of this setup do not permit motion in the z-direction, resulting in a constant positive moment around Mx. In the Toggling setup, both rotation and motion in the y-direction were constrained, leading to an alternating moment around Mx and displacement in the z-direction, with an almost constant Fz force.

To achieve loading conditions as physiological as possible, alternating compressive and tensile loads of ±110 N were applied in the Y-direction. The statistical analysis yielded reliable measurement results (Figures 3–5). In a previous study, Schmoelz et al. also applied alternating cranio-caudal loads starting at ±50 N, increasing by 5 N every 100 cycles up to a maximum of 600 N (Schmoelz et al., 2018). Based on *in vivo* measurements of pedicle screw loads, they concluded that this loading condition better represented physiological motion compared to a simple pull-out test. This hypothesis is supported by investigations from Rohlmann et al., who, using instrumented internal fixators in the spine, observed alternating loads during level walking (Rohlmann et al., 1997). Results from several previous studies, in which alternating cranio-caudal loading was applied, also produced more meaningful findings than pull-out experiments (Hirano et al., 1997; Dawson et al., 2003; Melkerson et al., 2003; Bostelmann et al., 2017; Schleifenbaum et al., 2025).

In contrast, Murray investigated three different loading cases under purely compressive loading and identified patient-specific loosening mechanisms (Murray, 2014). Murray described six partially overlapping failure mechanisms in the human vertebral body, including widening, cranial or caudal screw toggling, butterfly-type motion, as well as combined forms of toggling and widening. If we consider these mechanisms, the ASTM would primarily cause widening due to the Fz force. Toggling would result in a butterfly effect due to the alternating moment around Mx. However, these mechanisms usually do not occur in isolation, as radiological examinations have already shown (Dakhil-Jerew et al., 2009; Ko et al., 2010). Based on these loosening mechanisms and the correct combination of forces, moments, and bone substitute materials, physiological loading conditions in the body could be reproduced. Nevertheless, for further investigations, an alternative experimental strategy appears necessary to adequately capture realistic multifactorial interactions. Our investigations showed that the isolated load cases, ASTM and Toggling, do not cover the full range of physiological loads. Rohlmann et al. demonstrated that gait induces a complex load scenario in which moments and forces act simultaneously on the system, and thus do not represent an isolated loading condition (Rohlmann et al., 1997). In addition, studies (Schneider et al., 1997; Basaruddin et al., 2012; Oefner et al., 2022; Shah et al., 2025) examining the architecture and morphology of trabecular bone have demonstrated the bone’s anisotropic response to loads. These findings suggest that isolated ASTM F1717 and Toggling protocols do not adequately reflect the multidirectional demands placed on screw-bone interfaces. For this reason, a combined, multi-axial load would probably be the better option for a possible prospective study of the anchoring strength of screws. Depending on vertebral integrity, instrumentation, and applied load profiles, such an approach would elicit distinct specimen-specific loosening patterns. Importantly, a standardized methodology would facilitate patient-specific predictions of primary implant stability and allow for comparisons between different implants or surgical techniques under experimental conditions. One possible approach would be a multi-axial load application, following the concept of spine simulators as described by Wilke et al. (Wilke et al., 1994). Accordingly, it is necessary to examine whether established multisegmental approaches apply to individual spinal segments.

### 4.1 Limitations

The limitations of our study included the chosen foam blocks as a substitute for osteoporotic bones. Compared to the complex geometry of a vertebral body, a simplified cubic geometry was tested in this study, which, unlike human vertebrae, lacked both a cortical shell and a pedicle canal. This leads to limited cycles and exclusions of tested foam specimens. As a consequence of the exclusion, no loosening mechanism could be observed or compared across the load cases. For future tests, foam blocks with a cortical layer and pedicle might reduce failure in the screw-foam interface. Furthermore, friction cannot be eliminated when moving the xz-table, which could explain the noise of the curves under toggling load Fz and Mx.

### 4.2 Conclusion

The commonly used load cases for characterizing pedicle screws do not adequately represent the actual physiological loading conditions of the spine due to their isolated load application. In our view, reproducing these physiological conditions experimentally requires the application of combined, or mixed, loading modes. The forces and moments observed in this study can be meaningfully derived from the boundary conditions of both loading cases. Based on the findings of this study, we suggest a multiaxial load application to be more appropriate than the isolated load cases examined here. However, a method still needs to be developed and validated to combine these isolated load cases and reproduce clinically relevant failure mechanisms under controlled laboratory conditions.

## Data Availability

The raw data supporting the conclusions of this article will be made available by the authors, without undue reservation.
